# The Principles and Practice of Gynæcology

**Published:** 1885-05

**Authors:** 


**The Principles and Practice of Gynæcology.** By Thomas Addis Emmet, M. D., LL. D. Third edition, thoroughly revised, with one hundred and fifty illustrations. Philadelphia: Henry C. Lea's Son & Co. 1884.

The accomplished author states, in his preface to the third edition, that "so great is the advance and change of views during the past four years in gynæcology, that the preparation of this edition has necessitated almost as much labor as rewriting the volume. Every portion has been thoroughly revised, a great deal left out, and much new matter added." The first edition, which was published in 1879, has given a demand for the third edition, issued in the present volume. The work needs no recommendation to the profession from our pen. It has gained for its author and for the American profession more of well-merited praise and reputation than any other work. Indeed, it may be regarded as belonging to the classical medical literature of the present century. The present edition contains the views and experience of the author, in a form that has not been presented to the profession before, on prolapse of the vaginal walls; on lacerations at the vaginal outlet and through the sphincter ani and perinæum; on the methods of partial and complete removal of the uterus for malignant disease; on the surgical treatment of fibrous tumors; on diseases of the Fallopian tubes, and on diseases of the urethra. The chapters on these subjects contain about one hundred and seventy-five pages of new material. The work, therefore, is fully abreast the advances that have been made in this very important department of medical science at the present day. It contains the recorded experience of one of the most accomplished specialists in the world, and at once commands the favor of the profession, both in this country and abroad. We commend the work to our readers who are interested in the special subject of which it treats.

